# A Review on Acoustic Emission Testing for Structural Health Monitoring of Polymer-Based Composites

**DOI:** 10.3390/s23156945

**Published:** 2023-08-04

**Authors:** Noor Ghadarah, David Ayre

**Affiliations:** School of Aerospace, Transport and Manufacturing, Cranfield University, Cranfield MK43 0AL, UK; d.s.ayre@cranfield.ac.uk

**Keywords:** embedding, piezoelectric, PVDF, PZT

## Abstract

Acoustic emission (AE) has received increased interest as a structural health monitoring (SHM) technique for various materials, including laminated polymer composites. Piezoelectric sensors, including PZT (piezoelectric ceramic) and PVDF (piezoelectric polymer), can monitor AE in materials. The thickness of the piezoelectric sensors (as low as 28 µm—PVDF) allows embedding the sensors within the laminated composite, creating a smart material. Incorporating piezoelectric sensors within composites has several benefits but presents numerous difficulties and challenges. This paper provides an overview of acoustic emission testing, concluding with a discussion on embedding piezoelectric AE sensors within fibre-polymer composites. Various aspects are covered, including the underlying AE principles in fibre-based composites, factors that influence the reliability and accuracy of AE measurements, methods to artificially induce acoustic emission, and the correlation between AE events and damage in polymer composites.

## 1. Introduction

Monitoring the structure’s condition, i.e., health, is essential to ensuring failure is never reached in the structure’s lifespan. SHM can be defined as the ability to observe changes during the structure’s lifespan using a sensor. Three central components are typically needed to create an effective structural health monitoring system: a sensor network, incorporated hardware, and software [1]. The use of non-destructive testing (NDT) methods to evaluate damage inside structures has been well-developed. However, using NDT requires the presence of an inspector or for the structure to be dismantled. SHM will improve the structure’s safety dramatically due to continuous monitoring compared to the interval inspection by NDT. The shift has changed in the last 30 years to embed the sensor inside the structure, which will overcome NDT challenges [2], creating a smart material. Embedding the sensor within the structure will provide more accurate results due to how close the sensor is to the substructure [3]. 

A smart composite is a composite that has been modified either actively or passively. According to Fairweather [4], an active smart material is a material that can change its geometric or material properties under the influence of electric, thermal, or magnetic fields, thus having a built-in ability to transduce energy. Examples of active materials include shape memory alloys (SMA), piezoelectric materials, electrorheological (ER) fluids, and magnetostrictive materials. In contrast, a passive smart material is a material that lacks the ability to transduce energy. For example, an optical sensor can sense the signal but not actuate it. 

Rogers [5] defined smart materials as materials that can alter their physical properties in a particular manner when subjected to a particular stimulus. The physical change could be related to shape, viscosity, damping, or stiffness. Examples of stimuli include magnetic and electric fields, pressure, and temperature.

Different levels of damage assessment can be distinguished when assessing a structure: a qualitative indication of damage presence, damage location, damage size, and an estimation of the structure’s safety at this level of damage [6]. 

According to Cawley et al. [7], there are three reasons why the industry is slowly adopting SHM. Firstly, not enough attention is given to the business case, i.e., although the technology is available, the economic benefits might still be unclear. Secondly, the technology must be proven on an actual structure under realistic service conditions. Thirdly, it must be demonstrated that the technology needs to be robust, as it needs to provide understandable information and prove robust against different structures and load cases.

**Figure 1:**
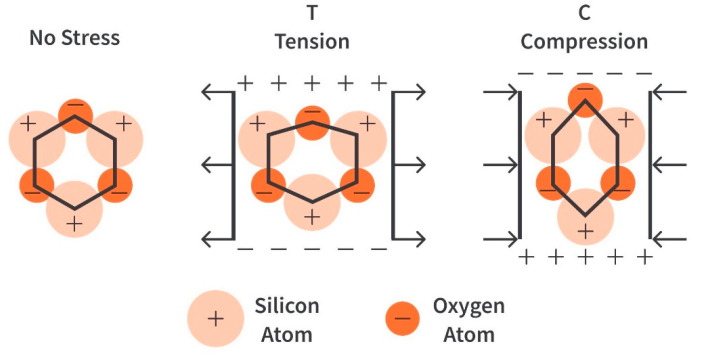
The piezoelectric effect in Quartz, reproduced with permission from [9].

Piezoelectric sensors are one of the vast number of sensors that can be used for SHM. Piezoelectric materials can induce an electric charge when mechanically deformed (direct piezoelectric effect) and mechanically deform when an electric charge is applied (inverse piezoelectric effect). The effect occurs because of the non-symmetrical crystal lattice of the material. Despite the non-symmetrical lattice, the crystal is still electrically neutral. As shown in Figure 1, atoms move when compressed or tensioned, altering the crystal and causing imbalance due to the position of the positive and negative atoms. The movement of the atoms will create two regions: positively charged dominant and negatively charged dominant [8]. The inverse piezoelectric material is based on applying a voltage to the piezoelectric, causing the crystal to realign its internal dipole structure, which will cause contraction and lengthening. This phenomenon causes electrical energy to be converted into mechanical energy. 

Four categories of piezoelectric materials can be identified: ceramics, single crystals, composites, and polymers. Composite piezoelectrics can be a combination of a single crystal or a piezoelectric material in a polymer matrix [10]. 

Piezoelectric materials can be used as receiving sensors and actuators due to their ability to induce a voltage when mechanically deformed and vice versa; i.e., they can be used for both active and passive sensing modes. Active sensing depends on the ability to send a signal from the sensor and measure the sent signal using either the same or a different sensor. In comparison, passive sensing depends on the ability to receive a signal without prior activation. Active acoustic monitoring can also be accomplished using irradiation-transient grating spectroscopy [11]. Active sensing commonly depends on a baseline measurement taken from undamaged structures to be later compared when in service [12]. Two active sensing methods can be used: electromechanical impedance and wave propagation [13]. Passive sensing, on the other hand, includes acoustic emission and stress wave propagation (impact sensing). However, the signal arising from the piezoelectric can change depending on the environment, including noise and temperature [14]. Hence, compensating for these effects is crucial.

## 2. Acoustic Emission (AE)

Acoustic emission is a naturally occurring phenomenon due to the sudden release of energy within the structure, resulting in a transient elastic wave [15]. Acoustic emission is considered a non-destructive testing (NDT) technique for ‘listening’ to the material. However, recent progress in SHM has increased interest in converting it into an SHM technique. AE works based on listening to the sounds produced by the structure when stressed or internally damaged, for example, when delamination or crack propagation occurs. AE equipment can detect sound frequencies ranging from 30 kHz to 1 MHz, with lower frequency ranges available if required. 

The acoustic emission output signal is usually confined to three types [16]: bursts, continuous, and mixed. Burst signals are transient signals that occur due to damage inside the material, including delamination and fibre breakage. In comparison, the continuous signal results from overlapping bursts where each burst cannot be individually identified. Mixed-signal, typically encountered in service, is where individual bursts and a continuous signal are present.

The type of failure occurring in a composite, whether delamination or fibre pull-out, can represent the current deterioration stage. Each type of failure has unique characteristics such as frequency, amplitude, rise time, and duration due to the distinct process that occurs at failure. That includes the crack tip’s motion, the crack surface area generated, and the crack propagation speed [17], as will be explained later. 

The acoustic emission technique allows the user not only to identify the failure type but also to locate the source of the crack generated. The time of arrival (TOA) difference can be used to determine the crack’s location. However, multiple sensors are required, and composites are mostly anisotropic, adding further complication, as later explained. Hence, one AE sensor is enough to detect damage but not enough to pinpoint the location of the damage. 

The threshold technique used by the acoustic emission system helps eliminate noise and allows for better readings. However, this technique will not always present the actual number of hits, as multiple transient signals can overlap, creating a strong burst (without dropping below the threshold) registered as one hit [16]. A better technique has been developed by Unnþórsson [16], which detects the hit start and end based on the trough and peaks of the signals. 

It is also preferable to use energy rather than the number of counts to analyse the magnitude of a source event. The energy is more sensitive to amplitude and duration and less sensitive to voltage threshold and operating frequency [18].

**Figure 2:**
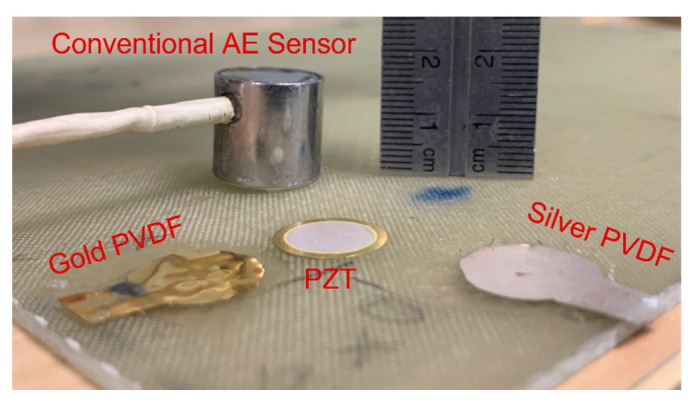
Comparison of the types of sensors applicable for AE testing.

Manufacturing composites does not always require high temperatures compared to metals, as low-temperature resins could be utilised. The thickness of the composite can also be controlled, enabling sensors to be embedded due to the manufacturing process. However, the size of a conventional acoustic emission sensor is too large to be embedded as seen in Figure 2. Regular piezoelectric sensors such as lead zirconate titanate (PZT) and polyvinylidene fluoride (PVDF) can be embedded in the structure due to their small size (gold-coated PVDF is available at 28 µm thickness [19]). Regular sensors are adequate but not covered by a shielding material, which is used to improve the signal-to-noise ratio of a piezoelectric sensor. AE sensors are typically placed on the surface of the specimen to be tested using a couplant, eliminating the acoustic impedance mismatch arising from the air. However, embedding the sensor will eliminate the need for a couplant.

### 2.1. AE Parametric Features

The parameters [20] used for AE analysis are listed and defined below:

**AE event**: The frequency domain or time domain of the acoustic emission signal emitted due to the elastic wave generation. The total AE wave during AE testing.

**AE hit**: An AE signal that crosses the threshold defined by the user from one channel, multiple AE hits can be generated due to multiple AE events or channels.

**Maximum amplitude**: The highest amplitude (voltage present; [21]) of an AE hit measured in decibels (dB) or voltage.

**Rise time**: The period from when the pulse first crosses the threshold and reaching the maximum amplitude.

**Counts**: The number of pulses of an AE hit greater than the threshold defined.

**Energy**: The area under the detection envelope within the AE hit duration.

**Duration**: The period between when the pulse first crosses the threshold until the last pulse threshold crossing

**Peak frequency**: The maximum frequency detected in an AE wave spectrum.

**Centre frequency**: The centre of gravity in an AE wave spectrum.

Average frequency:

(1) $$AF=\frac{Counts}{Duration}$$

**Initial frequency (IF)**: the AE spectrum’s initial condition. IF can be calculated by dividing the counts by the rise time.

(2) $$IF=\frac{Counts}{Rise\;time}$$

**RA Value**: It can be calculated by dividing rise time by maximum amplitude. It represents the type of crack (unit: ms/V)

(3) $$RA\;value=\frac{Rise\;time}{Maximum\;amplitude}$$

**Reverberation frequency (RF)**:

(4) $$RF=\frac{AE\;counts-counts\;to\;peak}{Duration-rise\;time}$$

**Figure 3:**
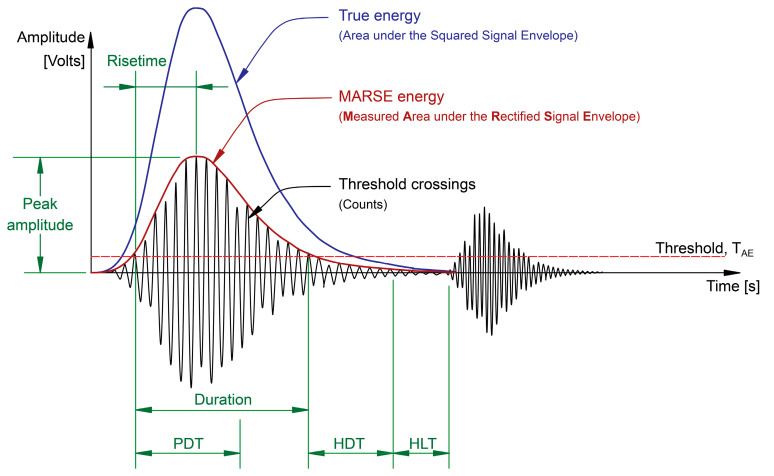
AE hit common parametric features extracted from hits, reproduced from [16].

Parameters used to determine the AE hits, shown in Figure 3 [16], are

**Peak definition time (PDT):** The amount of time allowed to identify the peak amplitude after the hit is registered (signal above threshold). Setting the PDT value too low or too high could cause false peak amplitude identification.

**Hit definition time (HDT):** The time specified after the signal becomes lower than the threshold to consider that a hit has ended. i.e., if the signal does not recross the threshold, the hit is considered to have ended. Setting the HDT value too low could prevent the whole hit from being captured and possibly treat one hit as multiple hits. Yet, if set too high, it may capture the next hit and treat two real hits as one hit.

**Hit lockout time (HLT):** The time that must elapse after a hit has ended before registering a new hit. If the HLT value is too high, a new hit can be missed, and if set too low, the system may capture late-arriving components or reflections as a hit.

### 2.2. AE Sources

The acoustic emission technique has been explored for implementation in composite structures by many industries, including aerospace [22], energy (wind turbine blades) [23], liquid hydrogen tanks [24], rocket motor casings [25], and automotive [26]. The approach is beneficial for the industry; however, the anisotropy within the composite poses a challenge with detecting failure events as the wave speed could differ based on the fibre direction, as will be discussed later.

#### 2.2.1. Composite Mechanical Loading

Different damage mechanisms can be analysed using the AE signals from fibre-matrix composites. AE signals emitted from fibre matrix composites can be divided into matrix cracking, fibre-matrix debonding, fibre breaking, and delamination [27,28]. Each damage mechanism has a unique acoustic (waveform) characterization [29]:

- Matrix cracking can be distinguished by low amplitude, low energy, and a slow rise time.
- Fibre-matrix debonding has a higher amplitude, higher energy, and shorter rise time.
- Fibre breakage has a quicker rise time, a short duration, a higher amplitude, and a higher energy.
- Delamination has a slow rise time, a much longer duration, and higher energy.

Knowing the failure type’s characteristics will enable the user to establish the danger level. That is, if the characteristics match those of fibre cracking, which is the structure’s load bearing, then the user can identify a critical danger level.

The average frequency response arising from the mechanisms is shown in Table 1. The frequency response registered by different authors is different due to the different fibre directions used, plies lay-up differences, final thickness, and AE machine used during the experiment. However, a general trend shows that the frequency response of matrix cracking < Fibre-matrix cracking < Fibre Fracture.

The magnitude-frequency graphs of the failure events have been identified by Asokan et al. [30].

The response of the damage mechanisms is also different when comparing the voltage-time graphs, as seen in Table 2.

**Figure 4:**
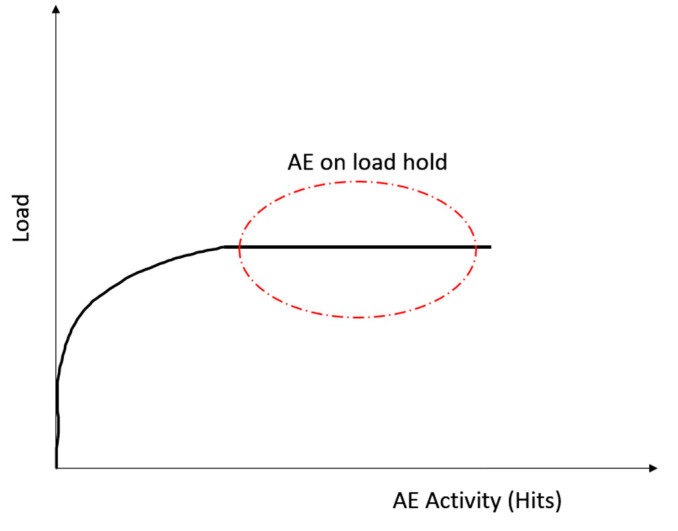
The acoustic emission with a constant load of an FRP, concept from [35].

In composites, acoustic emission is produced as expected when the material is under load. However, the acoustic emission may continue under certain conditions even at a constant load, as seen in Figure 4 [35]. The deformation can be delayed for several reasons. For example, in a fibre-reinforced polymer (FRP), the delay is due to the visco-elastic nature of glass fibres. Additionally, the structure’s stress history is particularly important since stress-induced deformation generates acoustic emission [36]. Certain materials take time to respond to load due to their intrinsic properties, such as the viscoelastic response in resin. Other materials produce AE almost instantaneously; upon holding the load, the emission stabilises. Other materials, such as structures with hydrogen-induced cracking, may never stabilise upon holding the load, causing continuous AE until failure [36].

The damage mechanisms, however, differ for sandwich-core composites [29]. Four types of signals can arise from sandwich core composites: resin cracking, fibres breaking, interfacial debonding between skin and core, and core damage.

- Core damage is characterised by a short rise time, a short time, and low amplitude and energy.
- Resin cracking is characterised by a slower rise time and higher energy and amplitude.
- Interfacial debonding waveforms have a slow rise time, a long duration, and a higher amplitude and energy.
- Fibre-breaking signals have a quick rise time, an extremely short time, and a very high amplitude and energy.

The characteristics of the AE waveforms can be correlated to a specific damage mechanism when applying simple loads. However, applying the AE technique to large structures can pose extra challenges due to the simultaneous activities occurring, leading to overlap or even signal misinterpretation. To demonstrate, a study performed on a full-scale aircraft fuselage composite panel showed that the high rate of failure within the composite is an added difficulty for detecting the damage source location [37].

**Figure 5:**
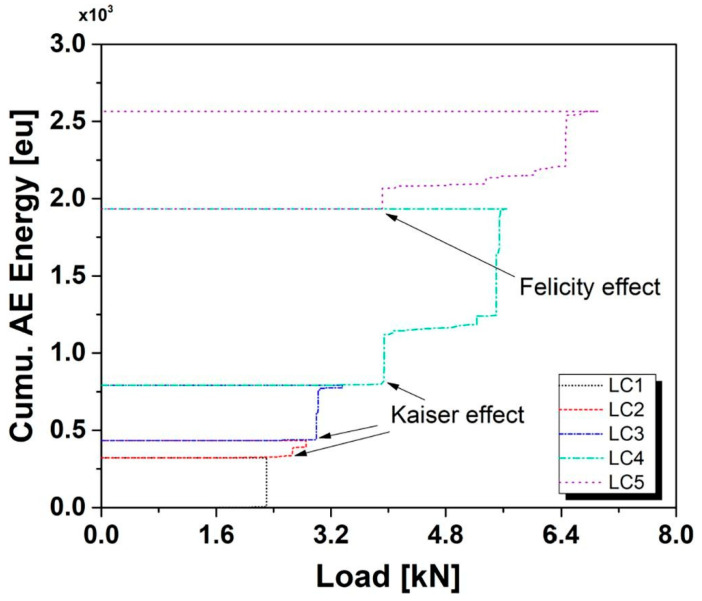
A graph showing the Kaiser effect, Felicity effect, reproduced with permission from [39].

Acoustic emission arises when the material is mechanically loaded (LC1, Figure 5). However, if the material is unloaded and loaded back up again, then no AE is recorded (horizontal line), which is known as the Kaiser effect [38]. The absence of AE is linked to damage already exerted from the first cycle, and no further damage occurs during the second loading. Upon loading the material (LC2, LC3 and LC4), new AE is detected due to damage occurring at higher loads than previously. Loading the material to higher loads produces significant damage compared to the low-level loads. Hence, upon unloading (end of LC4) and loading (LC5), the emission is exerted at a load lower than the maximum load exerted from previous cycles; the effect is known as the Felicity ratio (Equation (5)). The emission arising at previously reached loads is due to the cracks and their corresponding stress concentration areas that exhibit a local increase in stress with lower loads [17].

(5) $$FR=\frac{Load\;at\;which\;emission\;begins\;again}{previous\;maximum\;load}$$

The Kaiser effect takes place when the Felicity ratio is 1.0 or greater. That is when the load at which emission begins is equal to or greater than the previous maximum load [36]. Acquiring an FR of less than 1.0 can indicate that the structure has significant defects. 

#### 2.2.2. Physical Contact

Frictional noise that occurs due to mechanical contact between surfaces, such as the slip-stick mechanism, could result in rubbing or fretting, giving rise to the AE signal. Additionally, foreign objects impacting the structure, such as sand or rain, could produce a signal [40].

#### 2.2.3. Noise 

Noises arising from within the structure and the outside environment could give rise to false alarms or even cover integral signals, yet some are distinguishable and hence could be easily disregarded. External acoustic noise, besides structural vibration, does not heavily impact the acquisition system [20]. However, external rubbing and electrical noise can result in a continuous AE signal [16]. Internal rubbing between crack surfaces during damaged components’ loading and unloading phases produces an AE event as the crack opens and closes [40,41]. The noise in AE detection can be dealt with in multiple ways [36]. Firstly, narrowing the frequency upper and lower limits will eliminate signals arising outside the limits. Secondly, the noise could be eliminated from the source; if not, it could be eliminated by placing items such as damping materials in critical areas. Some electrical noise can be dealt with by proper grounding, shielding, or using differential sensors. If the noise cannot be dealt with directly, the AE software could be used to set a floating or fixed threshold that will ensure AE signals below a particular dB will not be detected.

Electromagnetic interference (EMI) and radio frequency interference (RFI) are produced from multiple sources, such as welding machines, fluorescent lighting, electric motor switches, and radio transmitters [40]. Pulse generators have also been identified as a source of EMI. Although it is challenging to eliminate EMI, it is typically associated with waveforms distinguishable from actual AE sources [17].

### 2.3. Artificial AE Sources

Multiple methods can be used to artificially induce an acoustic emission signal in samples, including pencil lead breaking, gas jet blasting, and transducer methods [42]. The methods induce an identical and reproducible signal, which enables the user to ensure the stability of the sensor, test attenuation, imitate a crack, or analyse different wave types. 

#### 2.3.1. Pencil Lead Breaking 

**Figure 6:**
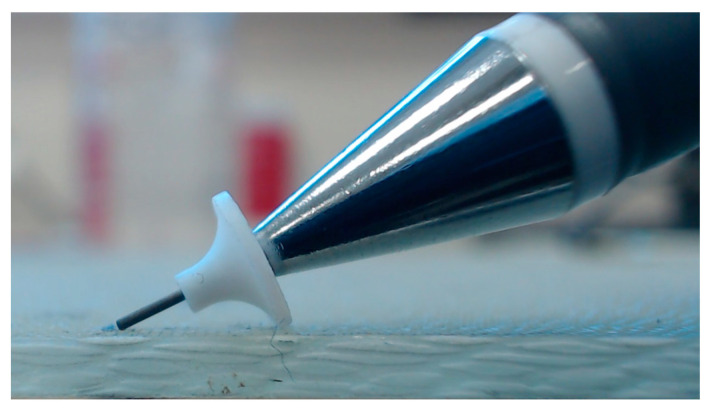
The pencil lead-breaking method.

The pencil lead breaking method, also known as HSU-Nielsen Source, is a method where a mechanical pencil is placed at an angle with the aid of a ‘shoe’ to be broken to generate an AE signal, as seen in Figure 6. The shoe is used to ensure consistency in the breaking angle with respect to the lead length (3 mm). Breaking the pencil lead will release the accumulated stress, causing the surface to displace microscopically, leading to the AE signal [43]. The test is widely used due to its availability, ease of use, portability, and avoidance of extra equipment. As the pencil must be broken at a certain angle, damage to the shoe’s ‘guide tube’ can lead to inaccurate results. The method does not allow for frequency control, which could be required in some instances. Acoustic emission testing aims to quantify the characteristics of emitted signals, such as amplitude, duration, and frequency content. However, pencil lead breaking may not provide sufficient control over these parameters, making it difficult to measure and analyse the data accurately.

#### 2.3.2. Gas Jet

**Figure 7:**
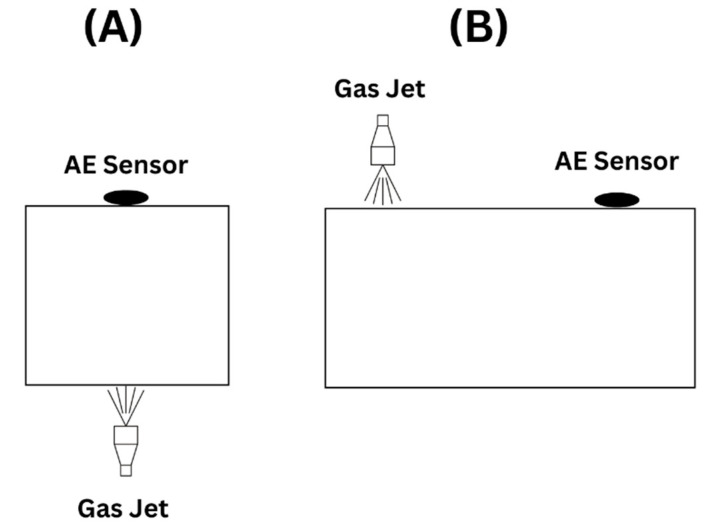
A Simplified drawing showing the gas jet method setup producing (**A**) longitudinal wave and (**B**) transverse wave, concept from [42].

The Gas jet method is less used than the pencil lead-breaking method due to the setup and equipment required for the test. However, the test is performed by permanently attaching a nozzle at one end and an AE sensor at the receiving end. A gas, which could be helium or dry air, is released at a recommended pressure of 150 to 200 kPa, inducing an AE signal. The test could be set up to induce a longitudinal wave, as shown in Figure 7A or a transverse wave, as shown in Figure 7B. The test requires damaging the structure by permanently attaching the nozzle to the test piece. In addition, removing and reattaching the setup for testing various locations is not convenient. Its requirement for high pressure poses a safety concern if it is not attached adequately compared to the other actuator methods.

#### 2.3.3. Transducer

An electrically driven transducer can generate an acoustic emission signal using either a white noise generator, sweep generator, or pulse generator [42]. Depending on the wave required, the transducer can be placed facing the sensor or on the same surface. When a white noise generator is used to power an ultrasonic transducer, an acoustic wave without coherent wave trains of many wavelengths at one frequency is created. In comparison, a sweep generator produces a signal with varying frequencies depending on the user’s input. For example, starting at 1 kHz and ending at 100 kHz for 10 s. A pulse generator, however, produces a pulse with a known rise and fall time, pulse width, and frequency. Although the transducer method requires extra equipment to operate, it can produce accurate results when permanently attached. The transducer method allows for frequency control compared to the other methods. However, the requirement for permanently attaching the sensor and the inability to remove and replace it pose a significant disadvantage.

### 2.4. AE Details in Composites

**Figure 8:**
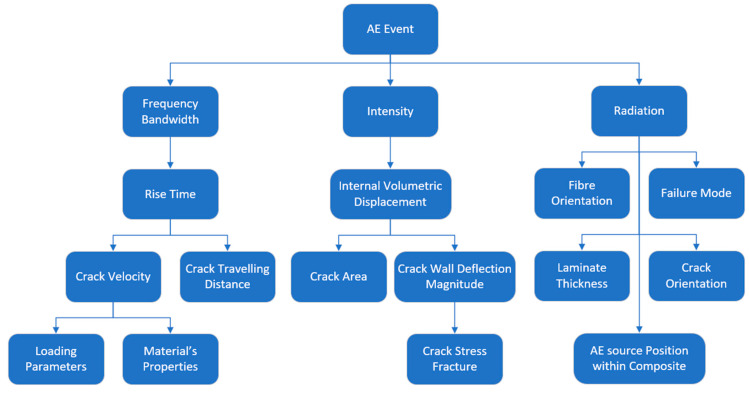
An overview of the acoustic emission details.

The acoustic emission source wave characteristics are linked to the dynamics of the crack [41]. That is, the crack growth speed of the source is linked to the material’s properties in addition to the loading parameters, which as a result, produces a specific frequency bandwidth. In addition, the failure mode and orientation of the AE source will create a distinctive radiation pattern. The position of the AE source within the composite could also influence the radiation pattern. Although different opinions were found regarding the intensity of the signal, Figure 8 shows an overview of the acoustic emission details according to the author’s opinion. 

#### 2.4.1. Bandwidth Response

**Figure 9:**
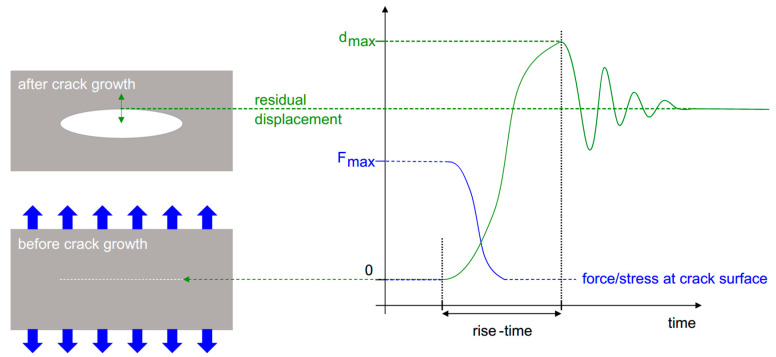
Crack growth with respect to the rise time, reproduced with permission from [41].

Acoustic emission sources occurring inside a material will produce a distinctive frequency bandwidth due to the dynamics of the source mechanism. The frequency bandwidth of the crack is directly related to the rise time of the crack [41]. As shown Figure 9, the rise time is the period required for the crack to reach its maximum displacement. However, once the crack has stopped (reached its final displacement), the displacement of the crack will not come to equilibrium due to its inertia, adding a contribution to the AE signal. 

Rise time depends on the propagating crack’s speed and the distance the crack will travel [41]. A fibre fracture will occur at a small distance of one fibre diameter and a high crack speed, producing a shorter rise time and hence high frequency bandwidth. However, matrix cracking occurs at a lower speed since the sound velocity in polymers is low [41] due to their lower Young’s modulus and higher density as per the general sound velocity in solids, Equation (6). Longer travel distances in polymers, when compared to fibres, increase the crack’s rise time and hence produce lower frequencies.

(6) $$v=\sqrt{\frac{Y}{\rho }}$$

where v is the sound velocity in solids and Y is the Young’s modulus of the material and ρ is the density.

#### 2.4.2. The Intensity of the AE Signal

According to Hellier [40], the amplitude and energy released from the acoustic emission source depend on the source event’s speed and size. A theory states that the AE amplitude is proportional to the crack’s velocity, and the AE energy released is proportional to the area of the new surface created. For example, a discrete, sudden crack will release high energy and produce a higher energy signal, whereas a creeping, slow crack will release less energy over the same distance.

**Figure 10:**
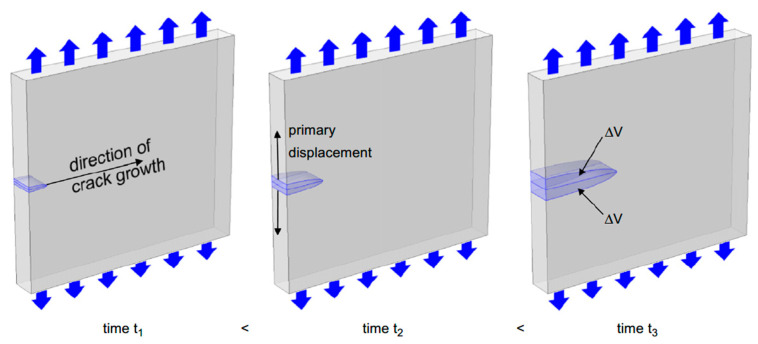
The process of forming the internal volumetric displacement, reproduced with permission from [41].

According to Hamstad [41], according to the generalised theory of acoustic emission [44,45], the intensity of the AE source is linked to the internal volumetric displacement (∆V) of the source. ∆V is linked to the crack’s area and the crack wall’s deflection magnitude, as seen in Figure 10 [41]. The crack’s wall deflection is related to the accumulated stress prior to the fracture. Materials with higher fracture stress will result in larger wall deflection, producing a more intense signal (higher AE amplitude). Rise time will also affect the signal’s intensity; a longer rise time will result in a more intense signal compared to a low rise time while keeping the fracture stress constant [41]. In contrast, Hellier’s theory [40] states that a discrete and sudden crack (indicating a low rise time) will produce a higher amplitude signal when compared to a creeping and slow crack (indicating a high rise time). 

The rise time will affect the signal’s frequency bandwidth, potentially causing the signal’s main intensity to be shifted to a frequency beyond detectable. A glass fibre in a polymer-reinforced composite has a higher fracture stress when compared to the resin. However, the displacement volume is much lower when compared to fibre/matrix cracking, which could extend throughout the composite, compensating for the strength and potentially matching the amplitude [41]. 

#### 2.4.3. Radiation through the Material

**Figure 11:**
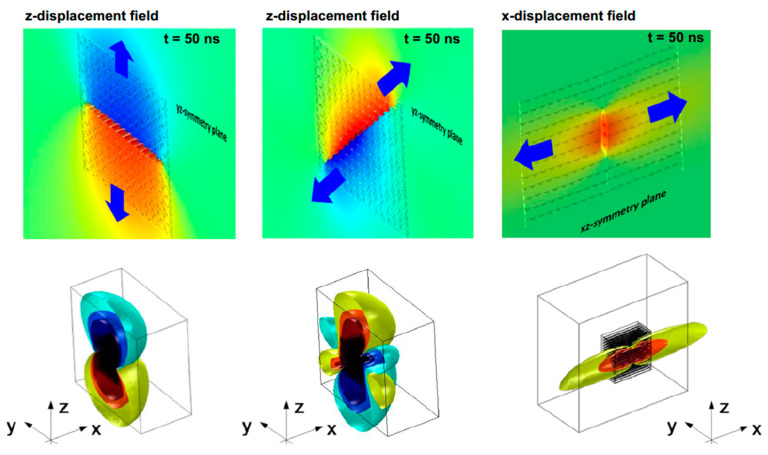
AE radiation patterns of (**left**) mode I interfiber failure mode, (**centre**) mode II interfiber failure, (**right**) mode I fibre failure, with the *z*-axis being the thickness of the laminate, reproduced with permission from [41].

The acoustic radiation pattern in composites is different from that of isotropic materials due to the thickness of the structure and the anisotropy associated with composites. For example, the AE event in metals is expected to spread evenly around the source, but that is not the case for composites, as seen in Figure 11. The fibre directionality in composites and the sound velocity difference between components affect the signal’s propagating path. Additionally, failures at the fibre/matrix interface or within the matrix material occur at arbitrary angles with complex superimposed stress states, meaning no unique radiation pattern can be identified [41]. 

**Figure 12:**
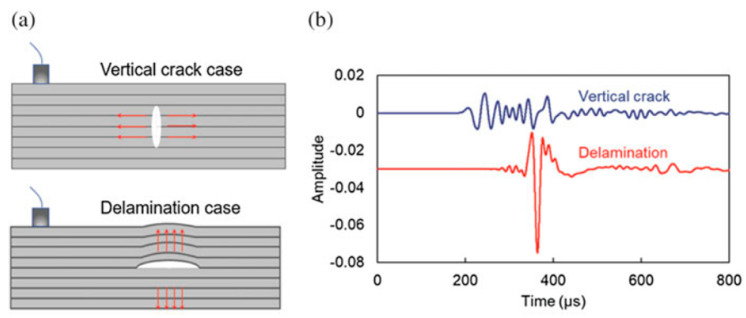
(**a**) Illustration of the crack orientation difference (**b**) signals originating from different crack orientations, reproduced with permission from [17].

The orientation of the AE source could also affect the shape of the waveform [17], as shown in Figure 12. That is, a vertical crack would produce different waveform characteristics when compared to a horizontal crack. The reason is that the vertical crack produces a mostly dominant symmetric wave compared to the horizontal crack, which produces an anti-symmetric wave due to the transient motion. Further details of the wave types are explained in 0.

### 2.5. The Signal-Shaping Chain

Four links have been identified to directly affect the shape and size of the measured signal: propagation of the sound wave, source, sensor and signal-conditioning electronics [35]. The signal shape between the source and the sensor is significantly different.

#### 2.5.1. Propagation of the Wave

The wave propagation inside a structure can be complex due to the surface geometry, which could reflect or deflect the wave. In addition, solid structures support compressional and shear forces, introducing numerous wave modes that can be simultaneously triggered. The two most important aspects of wave propagation are wave velocity and attenuation [35]. The wave velocity is essential to locating the source location compared to the attenuation, which determines the distances between sensors. It is also worth noting that information regarding the source mechanism is concentrated at the start of the waveform compared to the latter part, which is more influenced by geometry and could contain reflections [17].

#### 2.5.2. Wave Modes [35]

The waves emitted at the source location are bulk waves with two components, longitudinal and shear. Bulk waves are nondispersive, i.e., all waves have a constant velocity. Bulk waves attenuate quickly and can only be useful in very small specimens.

Other wave modes, such as Lamb waves and Rayleigh waves, are produced at the surface from a partial wave energy conversion. If the wall thickness is lower than one wavelength, Lamb waves are produced, but if the wall thickness is higher than one wavelength, Rayleigh waves are produced. Lamb waves are dispersive, meaning their velocity depends on the material thickness and frequency. 

**Figure 13:**
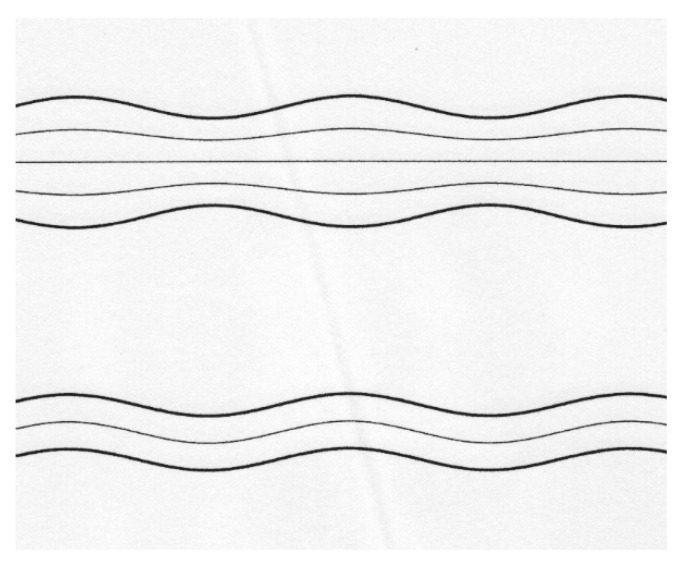
Lamb wave modes in plates, (**top**) Extensional (S_0_) and (**bottom**) Flexural (A_0_), reproduced from [46].

**Figure 14:**
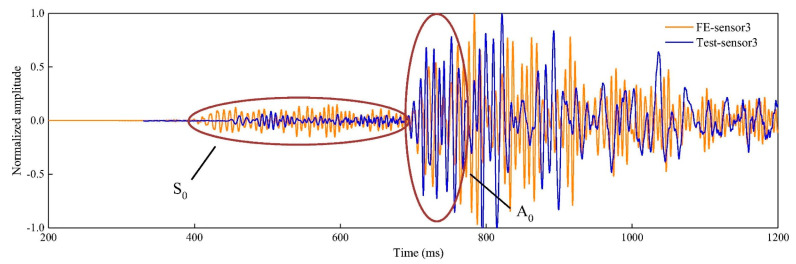
A representation of the AE waveform received by the sensor, reproduced with permission from [47].

Lamb’s theory identifies two wave modes: flexural, also known as anti-symmetric (a_0_ or A_0_), and extensional, also known as symmetric (s_0_ or S_0_), as shown in Figure 13. Other wave modes from the same family exist, but their amplitude is insignificant and hence can be disregarded. The two wave modes travel at different speeds. In a 0.17 cm thick steel plate at 100 kHz, the extensional wave travels at 4.7 km/s, whereas the flexural wave travels at 3.3 km/s. Hence, the extensional wave will reach the sensor before the flexural does. However, the flexural wave has a higher amplitude than the extensional wave, as seen in Figure 14.

Flexural mode (a_0_) is where the plate’s body deforms with the wave moving in the same direction. The motion is primarily transverse with very little longitudinal motion. The force applied needs to be perpendicular to the plate or parallel but offset from the centre to produce this wave mode.

Extensional mode (s_0_), or symmetric mode, is where the plate is alternately compressing and stretching in the wave direction. To produce this wave mode, the force applied has to be parallel to the plate or released by a rapid release of in-plane tension.

In thin plates, edge lead break preferentially produces the symmetric (S_0_) mode, whereas surface lead break preferentially produces the anti-symmetric (A_0_) wave [48]. 

**Figure 15:**
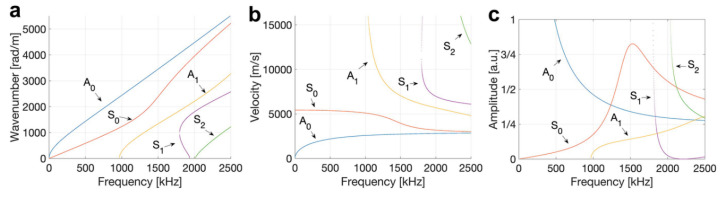
2 mm thick aluminium plate dispersion curves (**a**) wavenumber-frequency (**b**) velocity-frequency and (**c**) amplitude-frequency, reproduced with permission from [49].

The symmetric and anti-symmetric modes have also been identified to exist at different frequencies when excited. Different wave modes exist at different frequency ranges when producing guided waves, as seen in Figure 15. The structure’s thickness is also essential to consider. If the fundamental modes (A_0_ and S_0_) exist below 1 MHz-mm, then for a 10 mm-thick structure, the fundamental modes will propagate at below 100 kHz frequencies. In addition, note that the amplitude of the signal is also influenced by the excited frequency, as seen in Figure 15c [49]. Pant et al. [50] tested carbon fibre prepreg with a total thickness of 0.17 mm while placing PZT sensors and receivers at the surface. They found that the A_0_ wave is dominant at frequencies less than 100 kHz compared to the A_0_ wave, which was dominant from 200 kHz to 500 kHz, while both wave modes coexisted at frequencies between 100 kHz and 200 kHz.

According to the mathematical theory of “eigenfunctions”, wave modes travel independently and do not interfere with each other; i.e., multiple wave modes can travel simultaneously in the same material. At a given point, the motion can be defined as the sum of the motion of all the wave modes [35].

#### 2.5.3. Attenuation [35]

Attenuation in structures is defined as the loss of amplitude as the wave travels through. The attenuation curve is particularly essential for optimising sensor placement and allowing full structure coverage. Attenuation is caused by multiple factors: geometric spreading, absorption, and reflection (scattering). The influence of each factor is directly related to the distance between the sensor and the source event. If the distance between the sensor and the source event is close (a few cm), geometric spreading is the most influential. However, if the distance is large, absorption and reflection are the most influential. 

The geometric spreading effect can be explained as the sound wave attempting to travel in all 360° directions from the source event throughout the structure. The maximum change in wave motion and the static stress field is near the source event. Hence, the highest loss of energy occurs at the source event. In theory, the stress wave will propagate spherically and attenuate in a large-dimensional structure. In realistic structures, the structure’s dimensions will force the wave to propagate in a defined space, lowering the attenuation from the beam spreading. For example, a rod’s attenuation from geometric spreading is less than that of a concrete block.

The absorption of the wave by the structure itself causes attenuation. Polymer-based composites are more prone to attenuation due to their viscoelastic nature, which causes thermoelastic dissipation. The material through which the wave passes absorbs and converts elastic and kinetic energies into heat. At higher frequencies, the absorption of the wave is higher due to the shorter wavelength. One of the differences between the geometric spreading and the absorption mechanisms is that absorption is constant in the structure, whereas spreading is higher at the source. For example, if the structure absorbs 5 dB per cm, it will continue to absorb 5 dB per cm. In comparison, the decibel loss in geometric spreading is not linear.

Wave reflection that occurs at geometrical discontinuities and structural boundaries causes attenuation. Some wave energy is reflected when the wave encounters a discontinuity. Additionally, discontinuities can give rise to mode conversion. This effect is particularly essential for structures with complex geometry, which could have changes in connections, direction, stiffeners, and other boundaries between the source event and the sensor.

### 2.6. Couplant

The piezoelectric transducer signal can be affected by the medium between the structure and the transducer. Three main factors influence the signal: the transducer’s pressure against the material, the material’s surface, and the couplant [51]. The pressure applied to the AE sensor is crucial to achieving a good coupling condition. The pressure applied on a dry contact needs to be 0.7 MPa according to ASTM E650M–12 [52]. It was concluded that, generally, as mounting pressure increases, sensitivity increases. According to detect frequencies up to 2 MHz, 0.03 MPa (300 g/mm) is sufficient [51]. In comparison, more than 1 MPa (10 Kg/mm) is required to detect over 4 MHz [51]. 

A couplant material is needed between the surface-mounted sensor and the sample to obtain good signal strength and avoid signal reflection. A dry contact is highly undesirable, as the signal will weaken due to the air between the sensor and the specimen. The interface must be air-free as air has an acoustic impedance approximately five orders of magnitude smaller than the two touching surfaces [53]. To reduce the reflection of the AE, the couplant material’s acoustic impedance must be close to that of the tested material. A study was done by Theobald [53] to compare the couplants used and their influence on the signal. Multiple couplants were compared on a hemispherical aluminium block: silicon grease, ultrasound gel, propylene glycol, and glycerin. A dry contact was also tested. The couplant effect was recorded for both longitudinal and shear waves [53]. 

According to ASTM E650M–12 [52], it is best to ensure that the couplant is as thin as possible and that there should be no voids or air gaps in the couplant. Additionally, taper or unevenness in the couplant can reduce sensitivity. Furthermore, it is not recommended to use double-sided tape as a bonding agent. 

### 2.7. Piezoelectric-Based Acoustic Emission Sensors

#### 2.7.1. Piezoceramic Discs (PZT)

**Figure 16:**
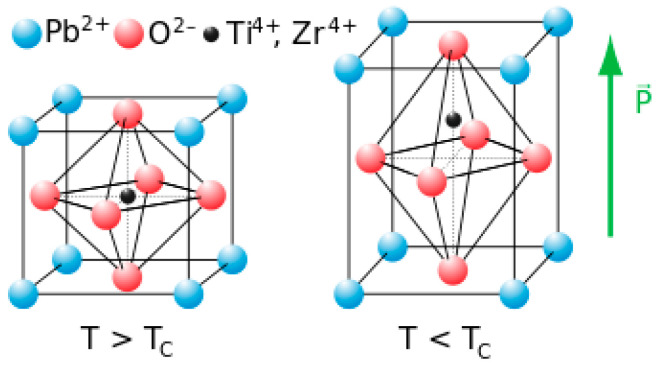
A schematic diagram showing a perovskite crystal structure where the PZT crystallites are (**left**) isotropic (centrosymmetric cubic) above curie temperature (**right**) anisotropic (tetragonal symmetry) below curie temperature, reproduced from [56].

PZT, or lead zirconate titanate Pb [Zr_x_Ti1 − _x_]O_3_, is a ceramic piezoelectric material. PZT has a perovskite crystal structure, where a small tetravalent metal ion (zirconium or titanium) is embedded in a lattice of large divalent metal ions (lead) in each unit [54], as shown in Figure 16. The crystal has a dipole moment if the structure is either tetragonal or rhombohedral. The PZT is made using multilayer technology where temperatures can reach 1100 °C; refer to [55] for more information.

#### 2.7.2. Polyvinylidene Fluoride (PVDF) Sensor

**Figure 17:**
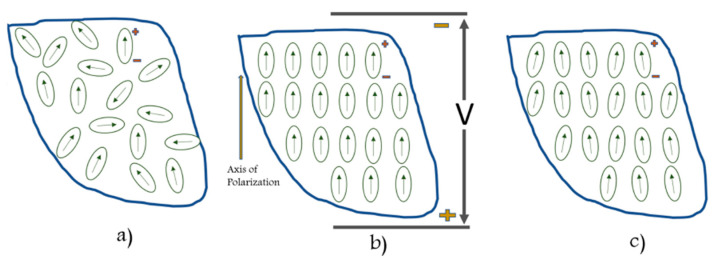
The polarisation process (**a**) domains are randomly distributed before polarising (**b**) Applying a large electric field across the structure to align the domains (**c**) after removing the electric field, the remnant polarisation persists, reproduced from [57].

Piezoelectric polymers can be divided into three categories: bulk polymers, voided charged polymers, and polymer composites [57]. One of the most widely used bulk polymers is Polyvinylidene fluoride (PVDF) because of its high chemical and mechanical stability, fast electromechanical response, low acoustic impedance, and flexibility. The piezoelectric effect in bulk polymers is mainly dependent on the molecular structure. Ferroelectricity largely depends on the material’s crystallinity, with amorphous polymers also having ferroelectricity due to the molecular dipoles in the amorphous region. A semi-crystalline material has an ordered structure (randomly oriented crystals) within an amorphous bulk. To enhance the piezoelectricity within the amorphous region, the material can be stretched to align the amorphous strands along the plane, which facilitates uniform rotation of crystallites when applying the electric field. The material must be poled just below the Curie temperature using a strong DC electric field to align the domains. The domains near the electric field would expand, causing a slight increase in length, usually in the micrometre range. Once the electric field is removed, the domains are locked into a near-alignment configuration, as seen in Figure 17.

**Figure 18:**
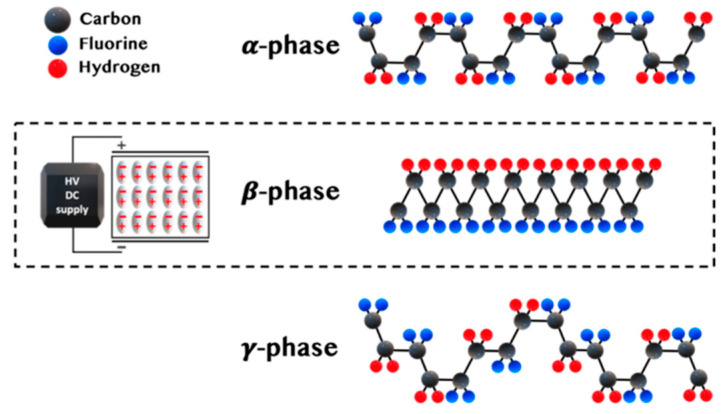
PVDF α-phase at the top, β-phase at the center and γ-phase at the bottom, where black atoms are Carbon, blue are Fluorine, and red are Hydrogen, reproduced from [59].

PVDF has multiple semi-crystalline phases that are a, b, g, d, and e, where a and b are dominant. The a phase is the most stable, and upon cooling from the melt, it develops a trans-gauche-trans-gauche semi-helical conformation. PVDF with a dominant a phase has zero net dipole moment due to the crystalline regions aligning such that dipole moments cancel each other. However, b-phase is the most suitable for piezoelectric applications as it is polar and electroactive. The b phase has a non-zero dipole moment due to the trans-conformation, meaning Fluorine and Hydrogen atoms exist on the opposite side of the main backbone chain, as seen in Figure 18. Thermal annealing, high-voltage treatment, and mechanical orientation can all be used to achieve the required crystalline phase transformation.

One of the methods adopted to create PVDF is solvent casting, where PVDF is dissolved in solvents like DMF or N-dimethylacetamide (DMA). The mixture is poured into a mould to create the shape and size desired, which can then be heated to remove the solvent. Spin coating can be used to achieve thin PVDF films.

The direction of stretching will change the behaviour of the PVDF (uniaxial or biaxial stretching). Stretching the material will result in an orthotropic material in the piezoelectric sense. However, the material is considered isotropic with low strains [58].

PVDF piezopolymers can be used for actuating and sensing applications. PVDF, as a sensor, is thought to be superior to piezoceramic because it is flexible (promoting easy formability), lightweight, and tough [60]. PVDF film size can be as low as 28 µm in thickness [19]. However, if PVDF is used as an actuator, it will produce a lower force than piezoceramic due to its lower modulus. PVDF property comparison with piezoceramic is presented in Table 3 [60], which shows the difference in the piezo activity (d_31_) and Young’s modulus between the PZT and PVDF films. Another advantage of PVDF is its ability to be cut to the required size and shape [61].

Multiple PVDF sheets are available on the market, one of which is a PVDF poled, gold coated on both surfaces, sold by Precision Acoustics [19]. An alternative is a piezo film sensor made up of rectangular piezo film elements with silver ink screen-printed electrodes. The maximum operating temperature of both sensors is 70 °C, further details in Table 4.

**Figure 19:**
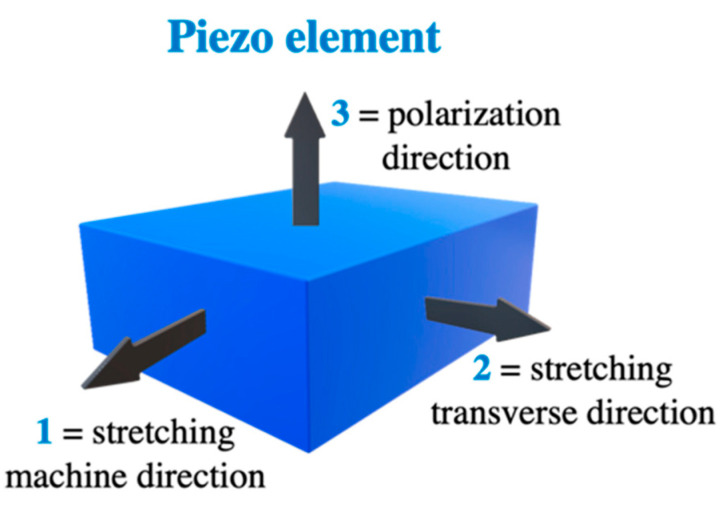
Piezoelectric material axis direction, reproduced from [59].

PVDF piezoelectricity is characterised by coefficients between the electrical and mechanical values [63]. The coefficients of the piezoelectric materials are formed based on the direction due to the anisotropic nature of the materials. The index of the value is $X_{ij}$, where i represents the direction of the electrical value measurement and j represents the direction of mechanical input. The numbers are based on the axis of the material (numbered from 1 to 3). As shown in Figure 19, numbers 1, 2, and 3 represent the machine direction, perpendicular (transverse direction), and through-thickness, respectively. For example, $X_{31}$ represents the electrical value on the 3rd plane and the mechanical input in the 1st plane. Most of the coefficients will start with i as 3 because the electrical electrodes are usually placed on top of the material.

Units of the piezoelectric coefficient to be considered, shown in Figure 19 [63]:

$d_{3j}$ (units: C/N) represents the film’s piezo activity. It corresponds to the electrical charge yielded from 1 $m^{2}$ when a 1 Pa stress is applied in the j direction. In addition, the mechanical strain when an electric field of 1 V/m is applied is denoted by (dLj/Lj). 

$g_{3j}$ (units: Vm/N) represents the electrical field supplied when a stress of 1 Pa is applied in the j direction.

g and d coefficients can be related using $g_{3j}=d_{3j}/\varepsilon .$

ρ (units: C/m^2^K) indicates the electrical charge density induced when the temperature increases by 1 °K.

Where C is coulomb, N is Newton, Pa is pascal, V is voltage, m is meter and K is Kelvin.

### 2.8. Fibre Optic Acoustic Emission Sensors (FOAES)

Optical fibres for AE sensing include various designs such as Mach-Zehnder interferometers, Michaelson interferometers, Fabry-Perot interferometers, fused-tapered couplers, and fibre gratings (FBGs) [64]. The dominant advantage of optical fibres over PVDF is their higher operating temperature. For example, the operational temperature for FBG AE sensing is reported to be between 25 °C and 200 °C [65]. Additionally, the performance of fibre-optic sensors is unaffected by electromagnetic interference [64].

#### 2.8.1. Fused Tapered Couplers

The technique is based on placing two optical fibres into parallel contact and then merging both using polishing, fibre fusion, or etching. When an AE event is triggered, stress waves interact with the fibres, which causes a change in the effective strain field, changing the two fibres’ coupling ratio’s output [64].

#### 2.8.2. Intensity-Modulated Fibre Optic Sensors

Intensity modulation is a method that uses a photodetector to measure the light intensity travelling through the optical fibre or reflected at the input. The lightwave intensity passing through the optical fibre can be altered by micro-bending the fibre, affecting the coupling between the fibres and the surrounding medium, or even breaking the fibre [66]. A study investigated damage monitoring in carbon-fibre reinforced polymers (CFRPs) using intensity-modulated fibre optic sensors [67]. The optical fibres will displace (micro-bend) due to the AE event. The detected AE event can then be analysed for possible matrix cracking, delamination, and fibre fracture. 

#### 2.8.3. Fibre Bragg Grating

Fibre Bragg grating (FBG) is an optical fibre with gratings in specific locations. The grating reflects a particular frequency depending on the spacing between the gratings. FBG can be used for strain measurement; when a structure is deforming, the spacing between the gratings will change, which will change the reflected frequency when compared to the undeformed state.

An essential aspect of detecting a crack or delamination in a composite is the overall structure’s strain change. The crack occurrence will only cause local strain (noticed at the crack tip), which is too low to be detected on the global strain field. In addition, if the fibre optic sensor, whether a line or point-distributed sensor, is not placed where the damage has occurred, the damage will not be detected [14]. 

Fibre Bragg grating can also be used for acoustic emission sensing by detecting the vibrations produced by the acoustic event [68]. However, the fibres can only detect vibrations along the fibre length, not perpendicular to the fibres. However, this can be enhanced using a cylindrical design where the fibres are wrapped around the cylinder.

### 2.9. Wave Velocity Inside the Material

#### 2.9.1. Experimental Calculation of Wave Velocity

Measuring the wave speed propagating inside the material is crucial for identifying the source location of a crack. Two sensors are required to calculate the wave velocity separated by a known distance (d). The sensors and the artificial AE wave must be aligned in a straight line [69]. The time of arrival for both sensors is registered and subtracted to find the time of arrival. The wave speed can then be calculated using the following formula:

(7) $$Velocity=\frac{Distance\;between\;sensor\;spacing\;(d)}{Time\;to\;arrival\;}$$

If the material is anisotropic, the wave velocity should be calculated in all directions.

#### 2.9.2. Theoretical Wave Velocity Inside the Material [70]

The plate wave theory calculates the wave velocity inside a material. While it is difficult to distinguish between the flexural and extensional wave modes, specific techniques, as previously mentioned, can preferentially induce either. To measure the longitudinal wave velocity ($C_{L}$) in an isotropic plate, the following equation is used:

(8) $$C_{L}=\sqrt{\frac{E}{\rho \left(1-\nu^{2}\right)}}$$

where E is Young’s modulus, ρ is density, and ν is Poisson’s ratio.

Whereas to measure the longitudinal wave velocity in an orthotropic laminated composite plate, the formula used is:

(9) $$C_{L}=\sqrt{\frac{E_{11}}{\rho \left(1-\nu_{21}^{2}\right)}}$$

where $E_{11}$ is the in-plane longitudinal elastic modulus in one direction, ρ is density and $\nu_{21}$ is the in-plane Poisson’s ratio in one direction.

Nevertheless, placing rectangular arrays of sensors in an anisotropic composite, for example, can provide an approximate location of the crack within the defined area of the sensors. Several algorithms/methods have been developed to determine the acoustic wave location (ASL) without taking into account the wave velocity, including, L-shaped sensor clusters [71,72,73], square-shaped sensor clusters [74], and Z-shaped sensor clusters [75], and the elliptic wavefront shape-based technique [76]. Special positioning of the sensors is necessary for the methods mentioned to operate effectively [76]. 

### 2.10. AE Source Location Identification Using Time of Arrival (TOA) [20]

Identifying the AE source location is a powerful tool that enables the user to identify the damage location. The simple method of calculating the source location is to know the time of arrival (TOA) and the wave velocity of each hit, therefore calculating the distance from the sensor. The ability to localise the damage is based on the delay of the incoming signal, i.e., the further away the source from the sensor, the longer it takes to arrive. However, an elastic wave travelling inside the material heavily depends on the propagation path’s properties and the propagation method to the AE sensor [20]. That is, different travelling effects, geometries (causing wave reflection), or intrinsic material heterogenous properties can cause imprecise wave velocity measurement. Hence, accurate wave velocity measurements are needed to provide the correct source location. 

Measuring the signal delay for composites is challenging as the fibres could act as a preferential path for the elastic waveform. In other words, a signal travelling through a fibre may reach its destination sooner than a signal travelling on resin.

Different techniques have been developed to identify the source location of the AE event depending on the number of AE sensors placed, as detailed in Table 5. 

#### 2.10.1. Linear Source Location Technique [20]

The linear source technique, also known as single (one) degree AE source location, is considered the most straightforward technique, requiring two AE sensors to determine the AE source location [20]. This technique is commonly used on pipes and bridges.

**Figure 20:**
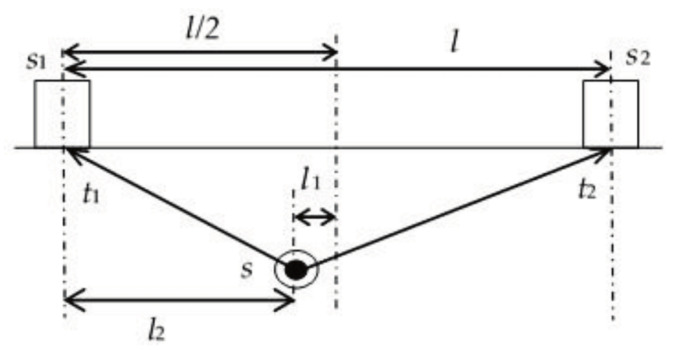
Linear source location schematic [20].

This technique depends on placing two AE sensors at a known and appropriate distance. The TOA from each sensor can be collected. The source location can be identified based on the difference in TOA between the two sensors. For example, if the difference in TOA between the two sensors is zero, then the source location is precisely centred between the two sensors. To further illustrate, the shorter the TOA, the closer the source location is to the sensor. Figure 20 shows a schematic of the technique, where s_1_ and s_2_ represent AE sensors. Furthermore, t_1_ and t_2_ represent the TOA to sensor 1 and the TOA to sensor 2, respectively. l, $l_{1}$ and $l_{2}$ represent the distance between the two sensors, axial distance to s_1_ and axial distance to the l/2 (midpoint between the two sensors), respectively. 

The source location can be calculated using the following mathematical relation, where Δt  represents the difference in TOA between sensor 1 and sensor 2 $\left(t_{1}-t_{2}\right)$ and v represents the AE wave velocity [20].

(10) $$l_{1}=\frac{1}{2}\left(t_{1}-t_{2}\right).v=\frac{1}{2}\Delta t.v$$

(11) $$l_{2}=\frac{1}{2}\;l-l_{1}=\frac{1}{2}(l-\Delta t.v)$$

#### 2.10.2. Two-Dimensional Source Location Technique [20,77]

This technique requires three or more sensors to identify the AE source location. The AE source will be surrounded by the three sensors, allowing the location to be more accurately measured [20]. Although three sensors are enough to identify the source location using the two-dimensional technique, a fourth sensor (often called a reference sensor) can improve the source location even further. The fourth sensor will allow a rectangular sensor array arrangement, producing six sensor pairs compared to three sensor pairs using three sensors. To achieve the two-dimensional AE source location, the TOA of each pair is calculated and correlated. 

### 2.11. Determination of the Time of Arrival (Onset Picking)

It is crucial to correctly determine the time of arrival of an acoustic wave to allow accurate determination of the source event’s location. To determine the time of arrival, the signal start must be identified. Multiple methods can be used to determine the TOA with varying complexity. The methods mentioned below were mainly used to detect seismic waves but can still be used for AE. 

#### 2.11.1. Amplitude Threshold Picker 

The simplest method to determine the TOA is to choose an amplitude threshold picker. This method allows the user to predetermine an amplitude threshold; whenever the wave amplitude crosses the threshold, the time of arrival is registered [18]. However, this method is unsuitable for high-noise or low-amplitude signals.

#### 2.11.2. Short-Time Average/Long-Time Average Ratio (STA/LTA) 

**Figure 21:**
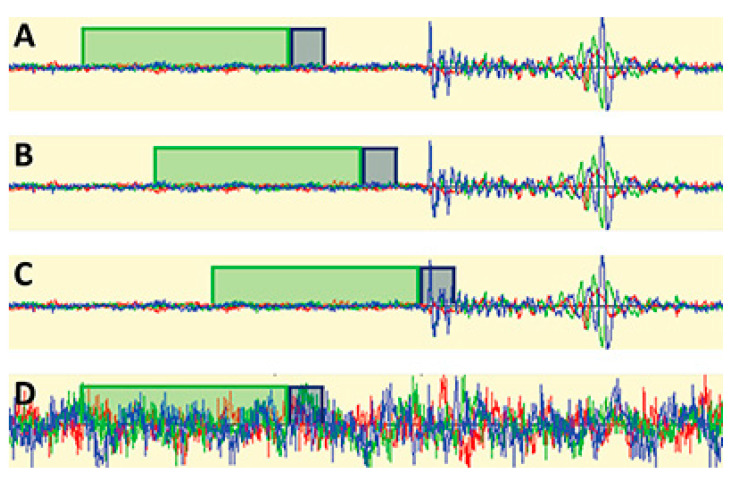
Green and grey boxes represent LTA and STA, respectively. (**A**–**C**) shows how the microseismic event is triggered (**D**) shows how false signals are not triggered, reproduced with permission from [78].

This method compares the short-time average amplitude (leading) to the long-time average amplitude (trailing). If the STA value is greater than the LTA value, then the STA/LTA ratio is greater than 1. A channel is triggered if the ratio exceeds the preset STA/LTA ratio threshold [78]. The process is better illustrated in Figure 21, where no channels are triggered if the average STA and LTA are similar (A,B). A channel is triggered if the STA has a higher value than LTA (C). However, in the presence of noise, the average values between STA and LTA are similar; hence, no channel is triggered (D).

#### 2.11.3. Akaike Information Criteria (AIC)

The AIC technique is based on the idea that the periods before and after a waveform’s arrival time are two separate stationary time series, each of which is regarded as an autoregressive process [79]. Multiple developments have been carried out in the AIC techniques, with one of the most recent improvements being made by Zhou [79]. 

### 2.12. Embedding Sensors

Embedding acoustic emission sensors is not limited to polymer composites as it has been tested within concrete [80,81] and with hip stem construction [82]. However, this section focuses on the application of embedding PZT and PVDF sensors in composites for acoustic emission detection. Embedding PZT and PVDF sensors are not limited to AE detection; instead, they could be used to detect strain by measuring the capacitance [83]. 

When integrating PVDF or PZT sensors inside a composite, the maximum operating temperature of the sensors is disadvantageously low. The low operating temperature of the sensors will limit not only the manufacturing technique used but also the matrix choice. In addition, mixing epoxy with a hardener can cause an exothermic reaction that releases heat, potentially damaging the sensor.

**Figure 22:**
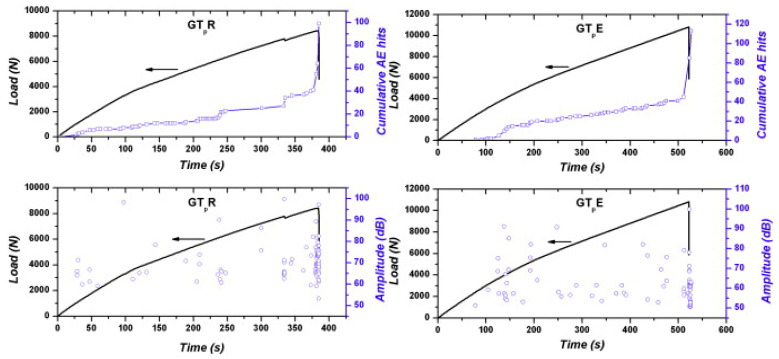
Cumulative hits and load (**up**) and amplitude distribution (**bottom**) for (**left**) sample with mounted sensor (**right**) sample with an integrated sensor, reproduced with permission from [61].

A study was also carried out by De Rosa [61] to identify the effects of integrating a PVDF with silver ink screen printed electrodes in an E-glass fabric/epoxy composite. The PVDF sensor size was 41 mm (length) × 15 mm (width) × 70 µm (thickness). The final specimen size was 200 mm (length) × 25 mm (width) × 1.8 mm (thickness). The study concluded from the tensile test that the modulus and the ultimate tensile strength of the samples with and without the sensor were within 6%. In addition, the flexural behaviour was similar, with a difference of 5% in modulus and 1% in bending strength between the samples. The number of AE events detected was slightly better with the embedded sensor when compared to the reference sample with surface-mounted sensors, as seen in Figure 22.

Masmoudi et al. conducted multiple studies investigating the influence of embedding sensors within composites [3,29,84,85,86,87,88]. The effect of placing a PZT sensor in an E-glass fibre composite was investigated [3]. Three different samples were prepared: a control sample with no sensors embedded (WS), a small sensor sample (SS), and a large sensor sample (LS) to be tested on a three-point bend test. The small sensor had a diameter of 5 mm and a thickness of 0.5 mm (SS), whereas the large sensor had a diameter of 10 mm and a thickness of 1 mm (LS). All the samples had a length of 150 mm, a width of 30 mm, and a thickness of 8 mm. The sensor was centred and placed 45 mm from the length edge. Compared to the controlled sample (WS), the failure load of the SS sample had a reduction of 8%, whereas the LS sample exhibited a decrease of 18%. On the other hand, the stiffness of the material remained the same, with a slight increase in the SS sample when compared to the controlled sample. 

**Figure 23:**
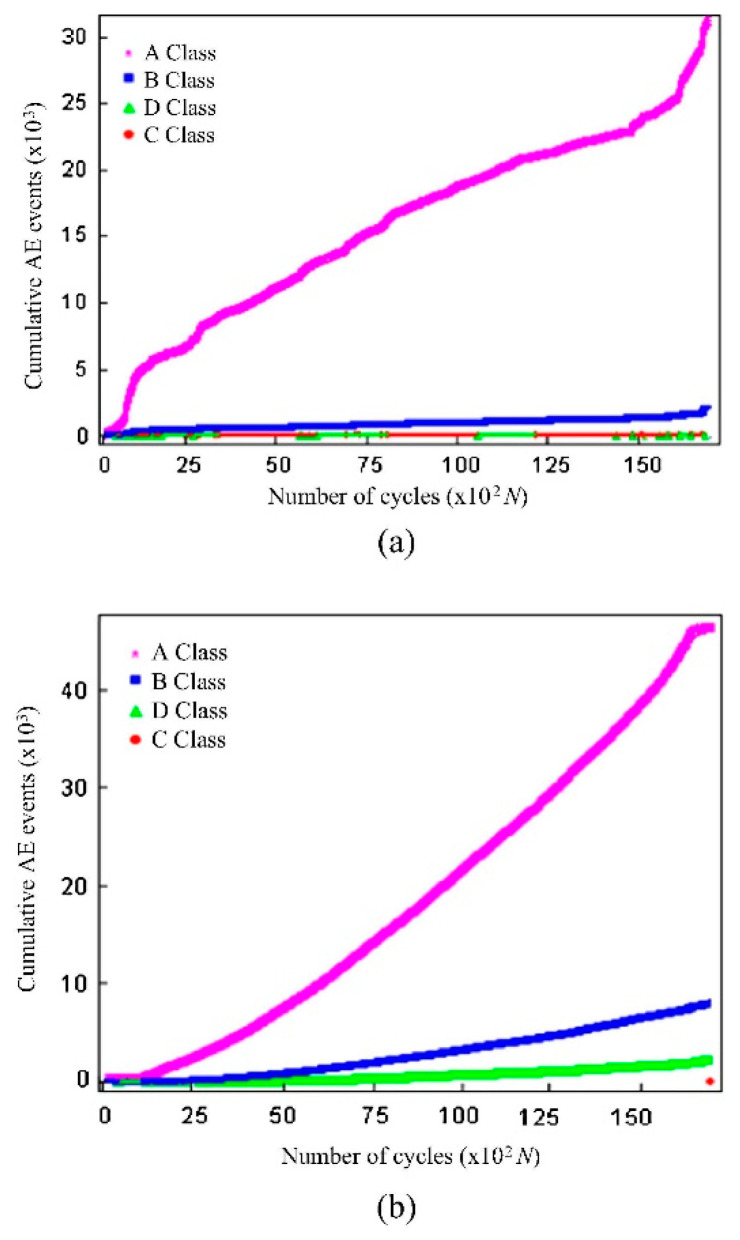
Cross ply laminate acoustic emission cumulative AE events difference between (**a**) specimen without integrating the sensor (**b**) specimen embedded with a small piezoelectric sensor [87]. A Class = matrix cracking, B Class = fibre-matrix debonding, D class = delamination and C = fibres breaking, reproduced with permission from [87].

As seen in Figure 23, embedding the sensor inside the composite has the advantage of being more sensitive, and hence more hits can be ‘heard’, resulting in more accurate results. In addition, there is no need for protective cladding to protect the sensor from the outside, as the structure itself will protect it. Theoretically, it should provide a better signal-to-noise ratio as the material surrounds it. However, it does have disadvantages, such as the inability to be integrated into existing structures, and if the sensor is damaged, false readings could arise. 

Another study [87] investigated embedding sensors in a cross-ply laminate [0_6_/90_6_]_s_, prepared using the hand lay-up process. The thickness of the laminate was 8 mm, whereas the sensor thickness and diameter were 5 mm and 0.5 mm, respectively. It was concluded that embedding the sensor caused minimal degradation to the mechanical properties and that the embedded sensor had higher sensitivity when compared to the surface-mounted sensor. A similar study on sandwiched composite [85] yielded similar results compared to the cross-ply laminate.

Syechal et al. [89] tested several sensor sizes with different fibre orientations in a natural fibre composite. The findings demonstrate that the inclusion of an insert increases fibre deviation and makes matrix-rich zones a preferred choice for crack initiation. However, the effect of the inserts on the samples differed based on the mechanical test performed. For example, the tensile test showed a reduction in strength, whereas with the three-point bend test, the insert did not affect the results and even occasionally improved them. However, the tensile-tensile fatigue resistance was affected negatively. The insert’s surroundings are affected by damage accumulating in the insert’s surrounding area, but the insert does not modify the mode or damage kinetics. 

Caneva et al. [90] have demonstrated that embedding the PVDF caused a slight mechanical strength reduction and that the embedded sensors were not more sensitive. Another study performed by Jain et al. [91] showed that increasing the number of glass fibre layers encompassing the sensor increases the sensor’s output voltage when testing using the lead break test. It also showed that the sensor with the lowest glass fibre layers yielded a higher voltage when performing an impact test until it fractured at 2 joules, but the sensor was still responding well.

Qing [13] investigated the ability to integrate a sensor network using piezoelectric sheets on a woven graphite/epoxy specimen. The thickness of the piezoelectric sheet is 0.15 mm. However, two different thicknesses of piezoelectric discs were used: 0.25 mm and 0.75 mm. It was found that the 0.75-mm disc got crushed once the mould was closed. Mechanical tests were conducted on a coupon specimen with a lay-up of ${\;[0}_{4}/90_{4}/0_{4}]$ an embedded piezoelectric sheet 0.15 mm in thickness and 0.25 mm piezoelectric discs. It was concluded that the sensor sheet defers delamination and does not noticeably affect the strength.

A recent study was done by Tuloup [83] to investigate the effects of embedding PZT and PVDF inside a glass fibre-reinforced polymer. Multiple tests were undertaken, such as monotonic tensile, load-unload, and cyclic loading. The embedded PZT and PVDF were used to compare the mechanical properties and investigate whether the embedding of the sensors causes a change in the acoustic emission response. When embedding PZT, the results showed that the mechanical property loss was negligible, with a 6% decrease in mechanical resistance (UTS) and a 5% reduction in Young’s modulus. However, the maximum longitudinal strain had increased by 11%, which caused a 26% reduction in Poisson’s ratio. At around 0.3% strain (y-direction), the PZT registered a slope change in the capacitance; upon inspection, cracks were found in the sensor. The acoustic response from the pristine and embedded samples was similar, confirming that embedding the PVDF does not change the damage mechanism. PZT had a different acoustic response due to the cracking of the PZT itself. It was found that there is a linear relationship between the capacitance and strain in both PZT and PVDF. However, the electrical capacitance variation for PZT, when compared to PVDF at the same strain, was much higher at 30% compared to PVDF at 0.7%. PVDF can receive signals until a sample fails, whereas PZT used to crack at 0.3% and hence become useless. One of the main disadvantages of PZT is its inability to withstand strain due to its ceramic nature. 

Ghezzo et al. [79] found that at least a four-layer laminate with a thickness of 1.05 mm is needed to integrate a 0.5 mm-thick PZT sensor. It was concluded that the thickness of the integrated device should be at least the same as the thickness of the laminates above and below it. 

A large sensor cannot be embedded into thin composites because the sensor is a rigid inclusion that can indent the matrix and damage the fibres [92]. Theoretically, the substantial decrease in mechanical properties can be reduced by increasing the composite’s thickness and reducing the sensor’s thickness.

## 3. Conclusions

The use of acoustic emission testing for structural health monitoring of polymer-based composite structures can be highly valuable in some instances while presenting significant challenges in others. The method can detect failure events and their locations without the need for activation. The crack location is challenging to determine in composites compared to metals due to their anisotropic nature, which leads to different wave speeds in different directions. Nevertheless, the effect of the anisotropy within the composite can be reduced using algorithms or special sensor placement. The current general trend towards artificial intelligence and deep learning will further enhance the AE technique, and more use of AE within the industry may be seen.

Extensive research has proven the ability to embed acoustic emission sensors in composites with partial disagreement. It has been established that embedding the sensors will affect the material’s mechanical properties depending on the sensor’s size and the material’s thickness. However, some research shows that embedding sensors improves their sensitivity, while others do not. The possible advantage of increased sensitivity can be more significant than the disadvantage of mechanical loss, depending on the application. The sensor can be placed in an uncritical location to reduce the effect of the mechanical properties drop.

The research conducted does not quantify the sensitivity of the sensors with respect to the location of the cracks. That is, whether a surface-mounted sensor is more sensitive to surface or sub-surface cracks or whether a fully embedded sensor is more sensitive to surface or sub-surface cracks. Previous work has focused on testing the acoustic emissions sensor’s sensitivity using mechanical testing, such as the three-point bend and tensile tests, where cracks could arise from the surface or sub-surface.

Calibrating the sensors and testing their attenuation requires a reproducible artificial AE source. The pencil lead-breaking method has been extensively used due to its ease of use. However, compared to actuators, its reliability on uneven surfaces, such as a peel-ply-induced surface, requires further investigation.

The protection of the sensor from the outside environment is not adequately explored in the literature. Environmental factors such as rain, sand, hail and debris impacts can cause interference or provide false results. For example, does embedding the sensor in a sandy environment prevent false signals from foreign objects? Or is it better to surface mount the sensor on the opposite side of the incoming foreign object? Any improvement in signal-to-noise ratio arising from embedding a sensor into a material has yet to be assessed and published. 

## Figures and Tables

**Table 1:** Failure frequency response of glass fibre/epoxy matrix composites.

Reference	Thickness (mm)	Frequency Response (kHz)			
		Matrix Cracking	Fibre-Matrix Cracking	Delamination	Fibre Fracture
[30]	2.78	90–110	-	130–200	250–280
[31]	4	62.5–125	125–187.5	-	187.5–250
[32]	5	100–190	200–320	-	380–430
[33]	4.8	60–180	190–250	-	350–500
[34]	4.8	11–93	82–210	-	160–281

**Table 2:** An overview of the responses acquired due to different damage mechanisms, reproduced with permission from [29].

Damage Mechanism	Form of Signal
Matrix cracking (A class)	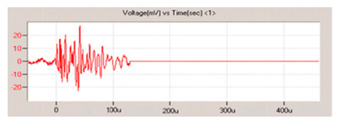
Fibre-matrix debonding (B class)	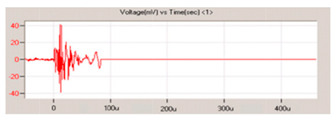
Fibres breaking (C class)	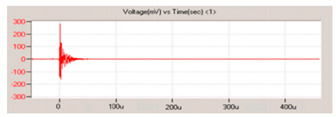
Delamination (D class)	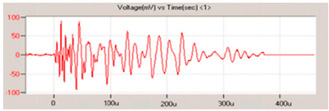

**Table 3:** Property difference between multiple piezo materials, reproduced with permission from [60].

Property	Units	PVDF film	PZT(PbZrTiO_3_)	BaTiO_3_
Density	kg/m^3^	1780	7500	5700
Relative permittivity	$\varepsilon /\varepsilon_{0}$	12	1200	1700
*d*_31_	10^−12^ C/N	23	110	78
*g*_31_	10^−3^ Vm/N	216	10	5
*k*_31_	at 1 kHz	0.12	0.30	0.21
Young’s modulus	GPa	~3	~60	~110
Acoustic impedance	10^6^ kg/m^2^-sec	2.7	30	30

**Table 4:** A comparison between the gold and silver PVDF.

	Gold PVDF [19]	Silver PVDF [62]
Thickness (t)	28, 52 and 110 µm	28, 52 and 110 µm (+12 µm for the protective film)
Maximum operating temperature (T)	70 °C	70 °C
Piezo strain constant ($d_{31})$	22 pC/N	23 pC/N
Piezo strain constant ($d_{32})$	3 pC/N	N/A
Piezo strain constant ($d_{33})$	−30 pC/N	−33 pC/N

**Table 5:** The source location technique to be used depending on the number of AE sensors [20].

Number of AE Sensors Required	The Dimension of the Source Location
Two AE sensors	Single (one) degree of source location
Three AE sensor	Two-dimensional source location
Four or more AE sensors	Three-dimensional source location

## Data Availability

The paper does not contain new data, rather it is a review.
